# Functional inertia reveals history-dependent organization of large-scale brain dynamics

**DOI:** 10.21203/rs.3.rs-9458803/v1

**Published:** 2026-07-13

**Authors:** Sir-Lord Wiafe, Vince D. Calhoun

**Affiliations:** 1 Tri-Institutional Center for Translational Research in Neuroimaging and Data Science (TReNDS), Georgia State University, Georgia Institute of Technology, and Emory University, Atlanta, GA 30303, USA.

**Keywords:** Functional inertia, Inertia state-space model (ISSM), brain dynamics

## Abstract

The brain does not process the present in isolation. Its responses are shaped by the accumulating weight of prior states, yet existing frameworks treat large-scale brain dynamics as sequences of transient configurations, leaving the constraining influence of history formally unspecified. Here we show that the brain possesses an intrinsic, representation-agnostic organizing constraint, the degree to which accumulated prior states resist ongoing reorganization, which we term functional inertia. Using an inertial state-space model replicated across two independent observational frameworks, we demonstrate that functional inertia operates coherently across three levels of brain organization: it structures activity into dynamical regimes, links these regimes to clinical and cognitive expression through a system-level inertial magnitude, and distributes across circuits in patterns that reconcile the longstanding coexistence of associative rigidity and sensory volatility in schizophrenia. Critically, removing the cumulative integration collapses this multilevel organization. Strikingly, the system-level inertial magnitude carries opposite cognitive signatures across diagnostic contexts, predicting better performance in healthy individuals but greater symptom severity in schizophrenia, reflecting context-dependent expression of a single constraint. These findings position functional inertia as a unifying, multilevel constraint on brain reorganization, recasting stability and volatility not as opposing properties but as context-dependent expressions of a single constraint.

## 1. INTRODUCTION

Large-scale brain dynamics are typically modeled as sequences of transient configurations, implicitly assuming that present activity is governed by instantaneous states^1–4^. Yet this assumption is incomplete: identical inputs can produce divergent neural responses not because the inputs differ but because the history of the system does^5–8^. Recurrent excitation^9,10^, NMDA receptor dynamics^9^, synaptic facilitation^11,12^, and neuromodulatory gain control^13–15^ together ensure that prior states persist and constrain how present activity evolves, yet none of the frameworks developed to characterize large-scale dynamics formalize this accumulated constraint. From dynamic functional connectivity and hidden Markov models^1–4^ to whole-brain generative models^16^ and predictive coding, activity is treated as sequences of transient configurations or learned distributional priors. The constraining influence of history on present reorganization remains implicit, assumed, and unmeasured across every scale of analysis.

This gap is not merely technical. The brain does not simply transition between states: it evolves under accumulated constraints imposed by prior activity^5–8^. Microcircuit models track moment-to-moment updates while leaving cumulative trajectories implicit^9,10^. Mesoscopic and electrophysiological approaches characterize evolving patterns but analyze state evolution relative to instantaneous configurations rather than accumulated context^6,17^. Even predictive coding and Bayesian brain theories, which most explicitly engage with the role of prior experience, formalize history as learned distributional priors rather than as a cumulative constraint on ongoing state evolution^18–20^. What is missing is not a better description of brain states but a formal account of how accumulated history governs resistance to reorganization, a quantity that is intrinsic, measurable, and scale-agnostic.

Here we show that the brain possesses exactly this constraint. We define functional inertia as the degree to which accumulated prior states resist ongoing neural reorganization and develop an inertial state-space model that treats accumulated context itself as a state variable, recursively integrating observations into a running latent trajectory whose evolution reflects the cumulative influence of prior states. Functional inertia is operationalized as the rate at which this accumulated trajectory changes, providing a quantitative measure of how strongly history constrains present reorganization. Crucially, the framework is agnostic to the form of observation and can be instantiated using spatial activity maps, regional or multivariate time series, dynamic connectivity trajectories, behavioral sequences, neuromodulatory signals, or any temporally evolving biological observable. Different observational axes provide alternative projections of the same underlying constraint rather than distinct mechanisms. Functional inertia is therefore not a property of any particular representation but an intrinsic constraint of how accumulated history governs state evolution across the full hierarchy of neural organization.

We instantiate this framework using dynamic time warping applied to resting-state fMRI, leveraging its natural cumulative alignment structure^21^ consistent with the temporal integration central to the ISSM. Functional inertia emerges as a coherent organizing constraint expressed consistently across three complementary levels of brain organization. At the dynamical level, it structures whole-brain activity into recurrent regimes reflecting distinct modes of history-constrained state evolution. At the system level, a stable inertial magnitude links these dynamics to cognitive performance and clinical symptom severity. At the circuit level, functional inertia is distributed across specific interregional pathways in patterns that differentiate normative and pathological dynamics. Using schizophrenia as a perturbative test case, a disorder characterized by well-documented disruptions in brain dynamics whose mechanistic basis remains contested, we show that departures from normative inertial organization emerge consistently across all three levels and are mediated by a shared system-level inertial magnitude, demonstrating that regime dynamics, global constraint, and circuit-level expression are coordinated manifestations of a single history-dependent organizing constraint.

Ablation analyses further establish the necessity of cumulative integration. Removing the accumulation step abolishes system-level inertial magnitude associations and mediation effects while preserving all other components of the model, demonstrating that accumulated context is not a redundant preprocessing step but the specific operation through which local regime dynamics cohere into a global organizing constraint. Consistent results across a correlation-based instantiation of the ISSM further indicate that functional inertia reflects a representation-agnostic constraint on brain dynamics rather than a modeling artifact. Together, these findings establish functional inertia as a unifying constraint on brain dynamics, one that operates across representational scales, coheres across levels of neural organization, and opens a new axis for understanding how accumulated history shapes both healthy cognition and psychiatric dysfunction.

## 2. RESULTS

### 2.1. Recurrent functional inertia states reflect whole-brain dynamical regimes

We instantiated the ISSM using dynamic time warping applied to interregional fMRI time series and defined the functional inertia index as the rate of change of the resulting accumulated latent trajectory, such that values near zero reflect strong resistance to reconfiguration and larger absolute values reflect active reconfiguration away from or toward a more cohesive amplitude configuration (Fig. 2a). To test whether FII exhibits structured dynamical organization, we applied k-means clustering to FII values from all interregional pairs and time points concatenated across subjects. The resulting regimes exhibited strong whole-brain segregation: within each regime, edges share the same sign and show a tightly concentrated range of magnitudes, indicating that these states reflect global modes of history-constrained evolution rather than distinct network configurations (Fig. 2b).

We identify three recurrent regimes. The locked regime, characterized by FII values near zero, reflects strong resistance to reconfiguration. The stabilizing regime, with positive FII values, reflects active reconfiguration toward a more cohesive amplitude configuration with decreasing interregional disparity. The shifting regime, with negative FII values, reflects reconfiguration toward a more dispersed configuration with increasing interregional disparity. Individual FII trajectories illustrate how subjects transition among these regimes over time (Fig. 2c). Together, these findings demonstrate that functional inertia organizes brain activity into three recurrent whole-brain dynamical regimes reflecting distinct modes and directions of history-constrained state evolution.

### 2.2. Altered engagement with functional inertia regimes in schizophrenia

Schizophrenia provided a perturbative test of whether functional inertia reflects a genuine organizing constraint: if accumulated history governs large-scale dynamics, disorders characterized by disrupted brain organization should exhibit systematic alterations in how inertial regimes are expressed and engaged. Group comparisons revealed significant diagnostic differences in both regime occupancy and persistence (Fig. 3a). Compared with controls, individuals with schizophrenia exhibited higher occupancy and longer persistence in the stabilizing regime, indexed by increased fraction rate (FR) and mean dwell time (MDT) (both p < 0.01). Conversely, controls showed greater occupancy and persistence in the locked regime, indexed by higher FR and MDT (p < 0.05). No group differences survived correction in transition probabilities.

Within the schizophrenia group, PANSS scores were primarily related to altered engagement with the locked and shifting regimes. Higher positive symptom severity was associated with stronger persistence in the locked regime, evidenced by increased self-transition probability, reduced transitions out of the locked regime, and elevated FR and MDT (all p < 0.05; Fig. 3b), and with reduced engagement in the shifting regime, including lower self-transition probability and lower FR (both p < 0.05). A similar pattern was observed for negative symptoms (Fig. 3c): higher negative symptom severity was associated with stronger persistence in the locked regime, evidenced by increased self-transition probability and reduced transitions out of the locked regime (p < 0.01) and higher FR (p < 0.01), alongside reduced engagement in the shifting regime, including lower self-transition probability (p < 0.001) and reduced FR and MDT (p < 0.05). Regime engagement metrics were not significantly associated with cognitive performance. These findings suggest that schizophrenia is not characterized by uniform disruption of inertial dynamics but by selective maladaptive persistence in high-inertia states, with symptom severity tracking the degree to which the system remains locked within accumulated configurations rather than engaging active reconfiguration.

### 2.3. System-level inertial magnitude carries opposite cognitive and clinical signatures across diagnostic contexts.

Beyond moment-to-moment regime dynamics, functional inertia is also expressed as a stable system-level constraint of brain dynamics, quantified as the time-averaged whole-brain functional inertia index magnitude (FIIM). FIIM is defined as the temporal mean of the absolute FII, such that lower FIIM reflects stronger overall inertia. Cognitive performance was assessed using the Computerized Multiphasic Interactive Neurocognitive System (CMINDS)^22^.

Among individuals with schizophrenia, lower FIIM was significantly associated with higher positive and negative symptom severity (Fig. 4a; p < 0.05), indicating that patients with more severe symptoms exhibit stronger global functional inertia. FIIM was also positively associated with processing speed, such that higher reconfiguration strength predicted better performance in this domain (p < 0.05). In healthy controls, the relationship inverted entirely (Fig. 4b): stronger functional inertia predicted better performance across processing speed, working memory, verbal and visual learning, reasoning and problem solving, and the composite CMINDS score (all p < 0.05). The same system-level inertial constraint therefore carries opposite functional signatures depending on diagnostic context, predicting cognitive resilience in healthy individuals and greater symptom burden in schizophrenia.

### 2.4. System-level inertial magnitude mediates the relationship between regime dynamics and clinical expression

Because regime engagement captures how the system-level inertial constraint is expressed over time, associations between locked-regime persistence and symptom severity should arise through the overall system-level magnitude rather than through regime timing per se. Mediation analyses confirmed this prediction: time-averaged whole-brain FIIM significantly mediated the relationship between locked-regime engagement and both positive and negative symptom severity within the schizophrenia group (Fig. 5). Greater locked-regime engagement, indexed by higher FR and MDT, was associated with lower FIIM, reflecting stronger overall functional inertia (path a; p < 0.01). Lower FIIM in turn predicted greater symptom severity after accounting for locked-regime engagement (path b; p < 0.05). When FIIM was included in the model, the direct associations between locked-regime engagement and symptom severity were no longer significant (path c’), indicating full statistical mediation. Bias-corrected bootstrap tests confirmed significant indirect effects for both positive and negative symptoms, with confidence intervals excluding zero.

To establish that this system-level organization depends specifically on cumulative integration, we performed an ablation in which the accumulation step alone was removed while preserving all other processing components (Supplementary Fig. 4). Although regime segmentation and several group effects remained, whole-brain FIIM associations and mediation effects disappeared entirely. Cumulative context is therefore not a redundant preprocessing step but the specific operation through which regime-level dynamics cohere into a global system-level organizing constraint.

### 2.5. Functional inertia is selectively distributed across circuits in patterns that track cognitive and clinical variation

To localize the system-level inertia associations, we examined relationships between time-averaged FIIM and cognition and symptom severity across 1,378 interregional brain network pairs derived using the NeuroMark template-guided ICA framework optimized for reproducible, standardized network estimation across datasets and disorders^23^.

In healthy controls, circuit-level FIIM showed domain-specific associations with cognitive performance (Fig. 6a). All significant associations showed negative coefficients, indicating that stronger circuit-level functional inertia predicts better cognitive performance. Working memory was linked to a focal sensorimotor-visual association pathway connecting the postcentral gyrus and middle occipital gyrus. Reasoning and problem-solving involved a broader distributed set spanning midline association cortex, frontoparietal control regions, visual association areas, and cerebellar pathways, including couplings between the precuneus and posterior cingulate cortex with occipital regions, anterior cingulate cortex links with middle frontal and middle temporal gyri, inferior parietal lobule connections with visual and sensorimotor cortices, and cerebellar couplings with occipital and parietal regions.

In schizophrenia, circuit-level FIIM showed widespread associations with symptom severity. For positive symptoms, 12 interregional connections survived FDR correction (Fig. 6b), with 10 edges showing negative regression coefficients indicating that stronger functional inertia is associated with greater symptom severity. These edges involved midline default-mode regions, including the posterior cingulate cortex and precuneus, limbic-thalamo-temporal pathways encompassing the hippocampus and superior temporal gyrus, and fronto-striatal and cerebellar links. The remaining two connections showed positive coefficients linking primary somatosensory and visual cortices to higher-order association regions. For negative symptoms, 29 interregional connections were significantly associated with PANSS-negative scores, with 24 edges showing negative regression coefficients. These connections spanned midline default-mode regions, including the anterior and posterior cingulate cortex and precuneus, temporal-sensorimotor interactions involving the fusiform and superior temporal gyri, and occipito-parietal-sensorimotor pathways linking the cuneus and superior parietal lobule with postcentral cortex and cerebellum. The remaining five connections showed positive coefficients concentrated in parietal and occipital association regions, including the bilateral inferior parietal lobule, paracentral lobule, calcarine cortex, and middle occipital gyrus. Together, these associations reveal a nonuniform inertial architecture in which distinct circuit classes show opposing relationships between inertia and symptom expression, with stronger inertia within associative control hubs predicting greater symptom burden and stronger inertia within sensory-association pathways predicting lower symptom severity.

## 3. DISCUSSION

Across recurrent dynamical regimes, a system-level inertial magnitude, and distributed circuit-level patterns, a single organizing constraint emerges: large-scale brain dynamics are organized not by instantaneous configurations but by the accumulated weight of prior states. Functional inertia formalizes this constraint, defining the degree to which history resists ongoing neural reorganization as an intrinsic, measurable, and representation-agnostic constraint on state evolution. This is not a description of specific brain states or connectivity patterns, but of the process by which they emerge and persist. By rendering this constraint observable and quantifiable, the inertial state-space framework reveals that stability, volatility, and clinical dysfunction are not independent phenomena but coordinated expressions of a single history-dependent constraint operating across levels of neural organization. Instantiated using dynamic time warping applied to resting-state fMRI and replicated across a correlation-based observational framework (Supplementary Fig. 3), this organizing constraint manifests coherently across three complementary levels of brain organization.

This organizing constraint is expressed dynamically through recurrent regimes of state evolution that reflect how strongly the brain resists reconfiguration at any given moment. Intuitively, a system governed by inertia cannot evolve arbitrarily^6^. It will necessarily pass through periods of minimal change, periods of directed movement toward a low-disparity context, and periods of movement away from it. In our data, these dynamics manifest as locked, stabilizing, and shifting regimes. Crucially, these regimes are not distinct spatial states or network configurations, but distinct modes of movement through state space under accumulated history. Their emergence as a three-regime organization reflects a directional inertial structure governing whole-brain evolution rather than isolated changes in connectivity patterns.

Functional inertia clarifies the organization of normative brain dynamics and how departures from this organization arise in schizophrenia. Healthy controls preferentially occupy the locked regime and exhibit greater persistence in this high-inertia state, whereas individuals with schizophrenia show reduced engagement of the locked regime and greater persistence in the stabilizing regime. At face value, this pattern could be misinterpreted as greater “stability” in schizophrenia and greater “rigidity” in controls. However, inertia is inherently contextual. High inertia is adaptive when the system resides within a well-organized, low-disparity latent context, where resistance to reconfiguration preserves a coherent configuration^16^. In contrast, stabilizing dynamics expressed on a disrupted latent baseline reflect constrained convergence within a limited dynamic range rather than restoration of a well-organized context. Consistent with this interpretation, regime-conditioned analyses show that locked regimes in controls occupy a significantly lower-disparity latent context than stabilizing regimes in schizophrenia, and that all control regimes reside closer to this context than any schizophrenia regime (Supplementary Tables 1 and 3). Thus, stabilizing dynamics in schizophrenia unfold on an already disrupted baseline and do not approach the latent context characteristic of healthy brain dynamics, consistent with prior fMRI and PET evidence of persistent functional disruption^24–26^. By separating the direction of reconfiguration from latent-state context, functional inertia provides a principled axis for interpreting why superficially similar dynamical regimes can carry opposite functional meaning. Rather than specifying which configurations occur, this axis constrains how readily the system can depart from established configurations.

When considered in relation to clinical expression, functional inertia suggests that schizophrenia is characterized not by excessive reconfiguration but by prolonged engagement of high-inertia states. Within the schizophrenia group, greater positive and negative symptom severity is associated with prolonged engagement of the locked regime, indicating that when the system enters a high-inertia configuration, it remains there. In this context, high inertia does not reflect adaptive stability but sustained residence within an already disrupted latent configuration. This interpretation aligns with prior dynamic connectivity findings showing that extended residence in weakly integrated or hypoconnected states predicts greater symptom burden^27,28^, but extends these observations by demonstrating that symptom severity is most strongly associated with persistence defined at the level of cumulative state evolution rather than instantaneous network patterns. From this perspective, clinical impairment reflects prolonged constraint within maladaptive latent configurations rather than transient fluctuations in connectivity states.

A striking feature of the system-level results is that the same inertial constraint carries opposite functional signatures depending on diagnostic context. In healthy individuals, stronger functional inertia predicts better performance across multiple cognitive domains, including processing speed, working memory, learning, and reasoning^29^, indicating that resistance to reconfiguration is adaptive when the brain operates on a well-organized latent baseline. In schizophrenia, the relationship inverts: stronger inertia predicts greater positive and negative symptom severity and poorer processing speed^3,28^, indicating that the same resistance becomes maladaptive when it operates on a disrupted baseline. This is not a paradox but a principled consequence of the inertial framework. Functional inertia does not have a fixed functional valence; its significance depends entirely on the latent context within which it is expressed. Resistance to reorganization preserves coherence when the system resides in a well-organized configuration and entrenches dysfunction when it resides in a disrupted one. The bidirectional pattern, therefore, does not reflect two different mechanisms but a single constraint expressed differently across two different contextual landscapes, resolving what would otherwise appear as a fundamental contradiction in the relationship between neural stability and cognitive function.

Because regime occupancy reflects the temporal expression of functional inertia rather than an independent mechanism, relationships between locked-regime persistence and symptom severity should arise through its overall magnitude. Consistent with this prediction, mediation analyses show that associations between prolonged locked-regime engagement and both positive and negative symptom severity in schizophrenia are accounted for by the time-averaged whole-brain FIIM. Greater locked-regime persistence predicted stronger global inertia, which in turn predicted greater symptom severity, and the direct association between regime engagement and symptom severity is no longer significant once global magnitude is included. These results indicate that regime dynamics and system-level inertia are not independent processes but complementary expressions of a shared constraint on state evolution. Within this framework, relationships between momentary regime engagement and clinical expression reflect how strongly accumulated history imposes global resistance to reconfiguration. Critically, this architecture depends on cumulative state integration. When the accumulation step is removed while preserving DTW alignment, smoothing, and derivatives (Supplementary Fig. 4), whole-brain FIIM associations and mediation effects disappeared. Thus, accumulation is not a redundant preprocessing step but the operation through which local regime dynamics cohere into a global inertial magnitude.

At the circuit level, functional inertia reveals a structured but nonuniform architecture of symptom expression in schizophrenia. Most symptom-related connections show an inverse relationship between inertia and symptom severity, such that lower inertia is associated with lower positive and negative symptoms. These associations are concentrated within distributed associative control hubs embedded within cortico–subcortical integration circuits, including midline default-mode regions such as the posterior cingulate cortex and precuneus, coupled with limbic, thalamo-temporal, fronto-striatal, and cerebellar pathways implicated in hallucinations, delusions, avolition, and social withdrawal^30–33^. This pattern suggests that stronger resistance to reconfiguration within integrative control networks is linked to greater symptom burden, consistent with reports of reduced variability in default-mode and control systems in schizophrenia^27,28^. In contrast, a smaller subset of connections shows the opposite pattern, with lower inertia associated with higher symptom severity, primarily involving sensory–association integration pathways linking primary sensory or sensorimotor cortices with higher-order associative regions. Prior electrophysiological and neuroimaging work indicates that effective sensory integration requires stable coordination between sensory and associative systems, whereas excessive volatility degrades perceptual fidelity and contextual gating^17,34^, consistent with dynamic connectivity findings in schizophrenia^27,35^. Together, these associations reveal a nonuniform inertial architecture in which distinct circuit classes show opposing relationships between inertia and symptom expression.

These circuit-level findings carry broader implications that speak directly to a longstanding debate in schizophrenia research. Prior work has produced apparently contradictory accounts: some studies emphasize reduced flexibility and over-stability in large-scale network dynamics^27,28^, while others highlight excessive variability and noise, particularly in sensory processing^17,34,36^. Functional inertia resolves this contradiction without invoking two separate mechanisms. Because inertia is context-dependent and circuit-specific, a single organizing constraint can simultaneously produce rigidity in distributed associative control hubs, where high inertia entrenches disrupted latent configurations, and volatility in sensory-association pathways, where low inertia degrades stable coordination between sensory and higher-order regions. Stability and volatility in schizophrenia are not opposing phenomena requiring separate explanations; they are context-dependent expressions of a single history-dependent constraint distributed nonuniformly across circuits^28,36–38^. This reframing positions functional inertia as a unifying account of why schizophrenia simultaneously exhibits hallmarks of both rigidity and noise, a coexistence that has resisted unified explanation until now.

Several considerations define the scope of the present analyses. Although the inertial state-space formulation is inherently representation-agnostic, and we illustrate this through an additional correlation-based instantiation (Supplementary Fig. 3), the primary analyses focus on a DTW-based formulation applied to resting-state fMRI as a principled example. This choice reflects one concrete instantiation among many possible observational axes rather than a restriction of the framework itself. We further adopt a zero-order instantiation with a uniform cumulative kernel, appropriate for resting-state conditions in which task timing and event structure are unspecified. This assumption does not imply that biological systems weight past experience uniformly; rather, it provides a minimal reference model against which more context-sensitive or adaptive accumulation rules can be evaluated. Extensions to task-evoked paradigms, longitudinal designs, alternative observational representations, and adaptive accumulation kernels would extend the same organizing constraint rather than modify its core formulation.

In summary, large-scale brain dynamics have a dimension that existing frameworks have left unmeasured: the degree to which accumulated history constrains present reorganization. This dimension is not incidental. It structures whole-brain dynamics into recurrent regimes, mediates the relationship between momentary dynamics and stable clinical expression, and distributes across circuits in patterns that explain longstanding contradictions in how schizophrenia disrupts neural organization. That a single quantity derived from accumulated context alone coherently organizes brain dynamics across all three of these levels suggests that history-dependent constraint is not a secondary feature of neural computation but a primary axis along which brain function and dysfunction are organized. Future work extending this framework to task-evoked paradigms, longitudinal designs, and other psychiatric conditions will determine how broadly functional inertia operates as an organizing constraint of neural and cognitive systems. What the present findings establish is that the brain’s present is never fully present: it is always shaped by the weight of where it has been.

## 4. METHODS

### 4.1. Inertial state-space formulation of brain dynamics

**Fig. 1:**
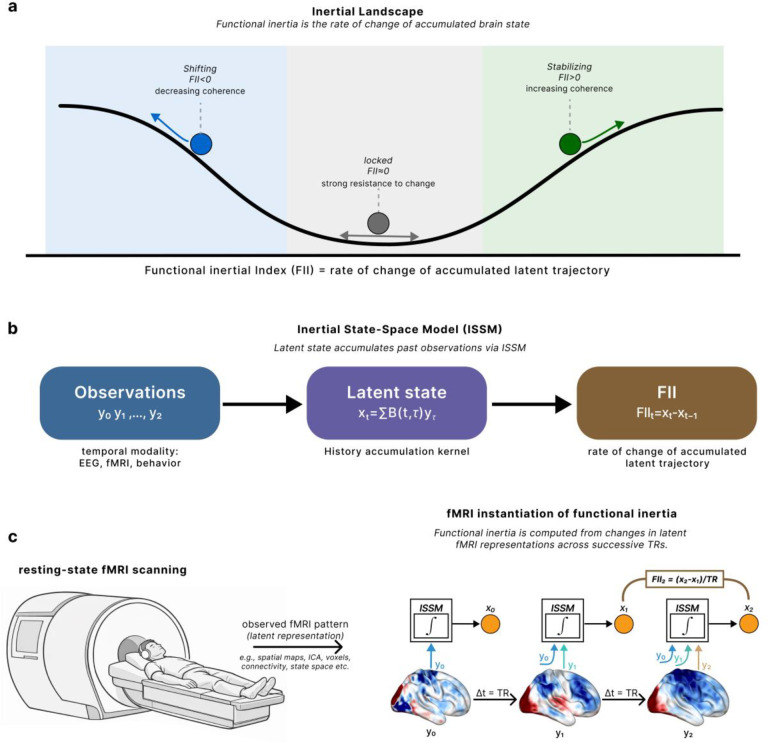
Functional inertia as a history-dependent constraint on brain state evolution. **(a)** Inertial landscape of functional inertia. Functional inertia is the rate of change of the accumulated brain state. The inertial landscape illustrates three recurrent dynamical regimes defined by the sign and magnitude of the functional inertia index (FII). In the locked regime (FII ≈ 0), the system exhibits strong resistance to reconfiguration. In the shifting regime (FII < 0), the system actively reconfigures toward decreasing coherence. In the stabilizing regime (FII > 0), the system actively reconfigures toward increasing coherence. **(b)** Inertial state-space model (ISSM). The latent state x_t_ is defined as a context-modulated accumulation of past observations y_τ_ through an accumulation kernel B(t, τ). The functional inertia index is operationalized as the rate of change of this accumulated latent trajectory (FII_t_ = x_t_ − x_t−1_). The formulation is representation-agnostic and can be instantiated across neural, behavioral, or physiological observables. **(c)** fMRI instantiation of functional inertia. fMRI observations are mapped to latent representations across successive repetition times via the ISSM. Each successive integration step accumulates prior observations, and changes in the accumulated latent trajectory are used to compute the functional inertia index at each time point.

**Fig. 2:**
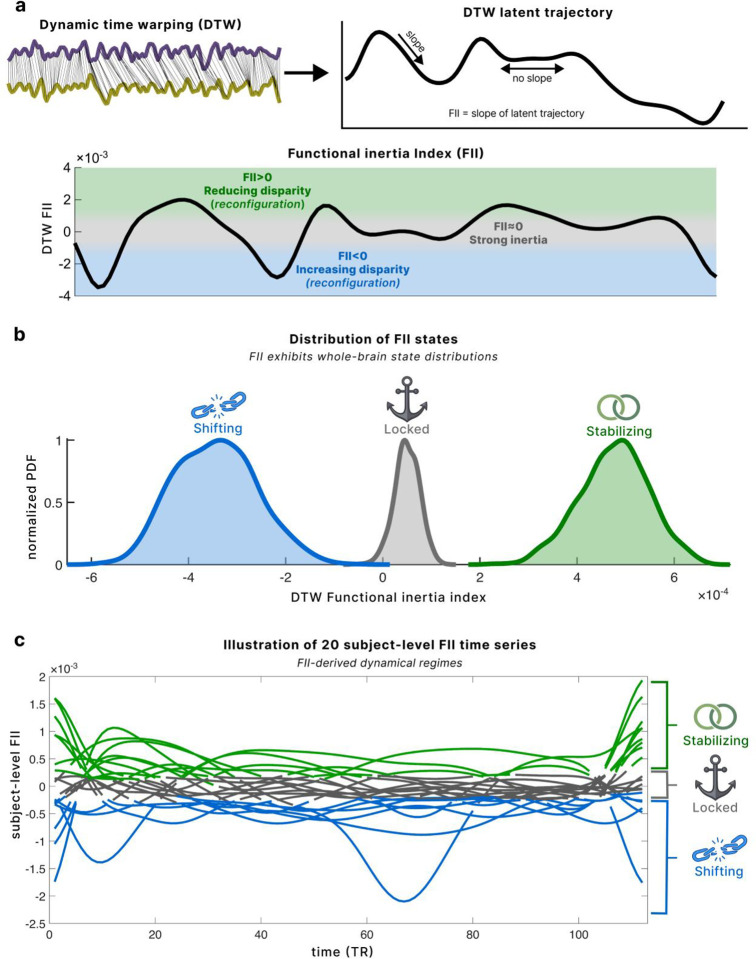
Dynamic time warping instantiation of functional inertia and emergent dynamical regimes **(a)** Dynamic time warping (DTW) aligns interregional amplitude trajectories while accommodating intrinsic timescale variability, yielding a time-resolved deviation signal. This deviation is recursively accumulated to form a latent trajectory that reflects the integrated history of prior observations. The functional inertia index (FII) is defined as the temporal rate of change of this accumulated trajectory. The functional inertia index is defined as the temporal rate of change of this accumulated trajectory, with values near zero reflecting strong resistance to reconfiguration and nonzero values reflecting active reorganization. **(b)** Distribution of functional inertia states across the whole brain. Pooling FII values across all subjects, time points, and interregional pairs reveals a trimodal distribution corresponding to three distinct dynamical regimes. These regimes are centered around negative FII values (shifting), near-zero FII values (locked), and positive FII values (stabilizing), indicating that functional inertia organizes brain dynamics into qualitatively distinct modes of state evolution. **(c)** Subject-level functional inertia time series illustrating recurrent dynamical regimes. Representative FII trajectories from 20 individuals show that these regimes recur over time within individuals and are expressed coherently across interregional pairs.

**Fig. 3:**
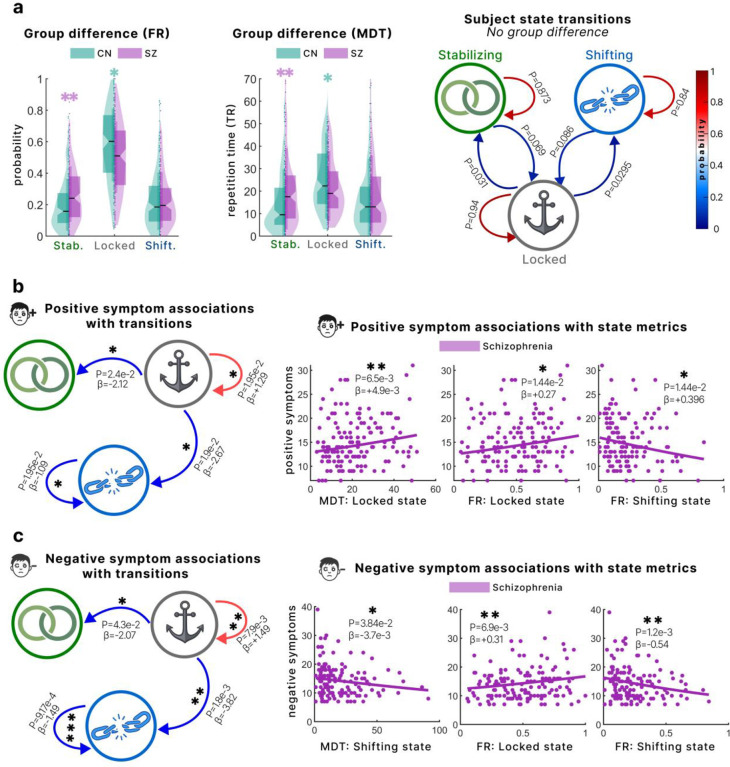
Engagement with functional inertia regimes across health and disorder **(a)** Group differences in functional inertia regime engagement and transition structure. Left and middle panels show group comparisons between healthy controls (CN) and individuals with schizophrenia (SZ) for fraction rate (FR) and mean dwell time (MDT) across stabilizing, locked, and shifting regimes. Individuals with schizophrenia exhibit increased occupancy and persistence in the stabilizing regime and reduced engagement of the locked regime, whereas no significant group differences are observed in state transition probabilities (right). **(b)** Associations between positive symptom severity and functional inertia dynamics in schizophrenia. Left, state-transition associations show that higher positive symptom severity is linked to reduced transitions out of the locked regime and reduced engagement of the shifting regime. Right, regression analyses demonstrate that greater persistence (MDT) and occupancy (FR) of the locked regime are associated with higher positive symptom severity, while greater engagement of the shifting regime is associated with lower symptom severity. **(c)** Associations between negative symptom severity and functional inertia dynamics in schizophrenia. Left, transition analyses reveal that higher negative symptom severity is associated with increased self-persistence of the locked regime and reduced transitions involving the shifting regime. Right, higher negative symptom severity is associated with increased occupancy of the locked regime and reduced engagement of the shifting regime.

**Fig. 4:**
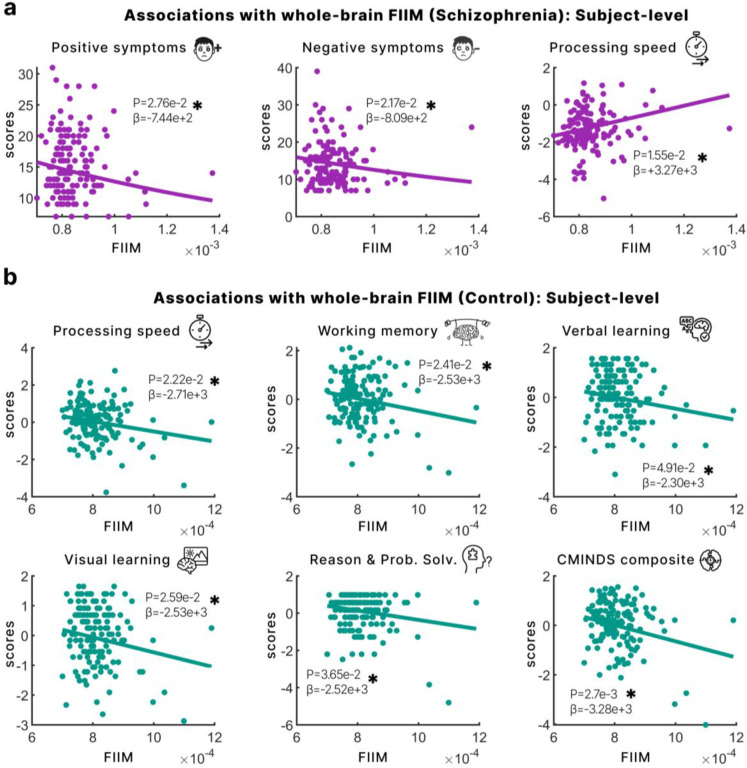
Whole-brain functional inertia magnitude differentially relates to cognition and symptoms across health and disorder **(a)** Associations between whole-brain Functional Inertia Index Magnitude (FIIM) and clinical and cognitive measures in individuals with schizophrenia. Lower FIIM, reflecting stronger overall functional inertia expressed across time and interregional pairs, is associated with greater positive and negative symptom severity and with poorer processing speed. Each point represents a subject; lines indicate fitted regression relationships after covariate adjustment. **(b)** Associations between whole-brain FIIM and cognitive performance in healthy controls. In contrast to schizophrenia, stronger functional inertia (lower FIIM) is associated with better performance across multiple cognitive domains, including processing speed, working memory, verbal learning, visual learning, reasoning and problem solving, and the composite CMINDS score. Together, these results show that functional inertia is expressed as a stable, system-level constraint on brain dynamics with dissociable functional consequences depending on the underlying latent-state context.

**Fig. 5:**
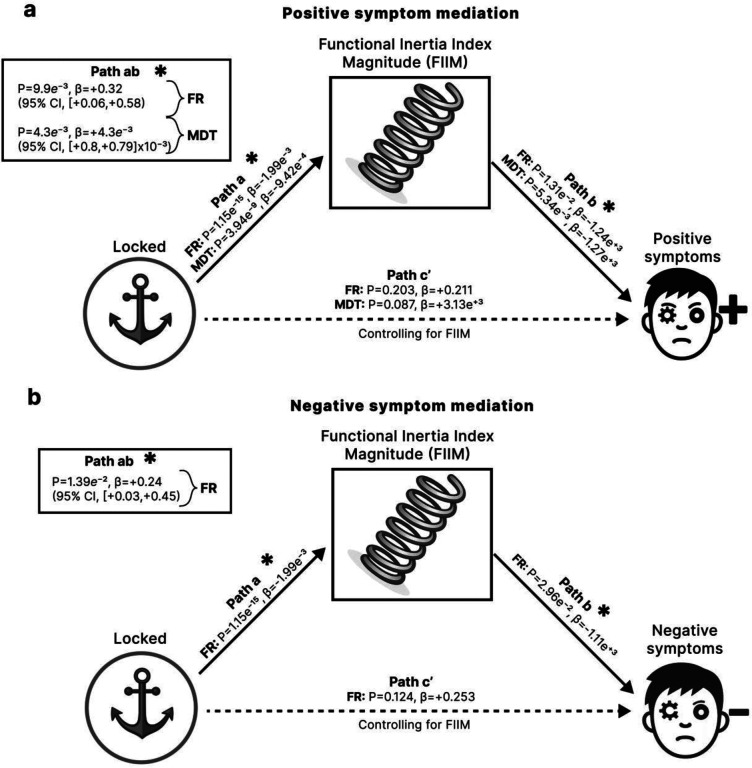
System-level functional inertia mediates the relationship between inertia regime engagement and symptom severity **(a)** Mediation analysis for positive symptom severity in schizophrenia. Greater engagement of the locked functional inertia regime, quantified by fraction rate (FR) and mean dwell time (MDT), predicts stronger whole-brain functional inertia index magnitude (FIIM) (path a). Stronger functional inertia in turn predicts greater positive symptom severity (path b). When controlling for FIIM, the direct association between locked-regime engagement and positive symptoms is no longer significant (path c’), indicating full statistical mediation. **(b)** Mediation analysis for negative symptom severity. Engagement of the locked regime, indexed by FR, is associated with whole-brain FIIM (path a), which in turn predicts negative symptom severity (path b). The direct association between locked-regime engagement and symptoms is no longer significant after accounting for FIIM (path c’), indicating full statistical mediation.

**Fig. 6:**
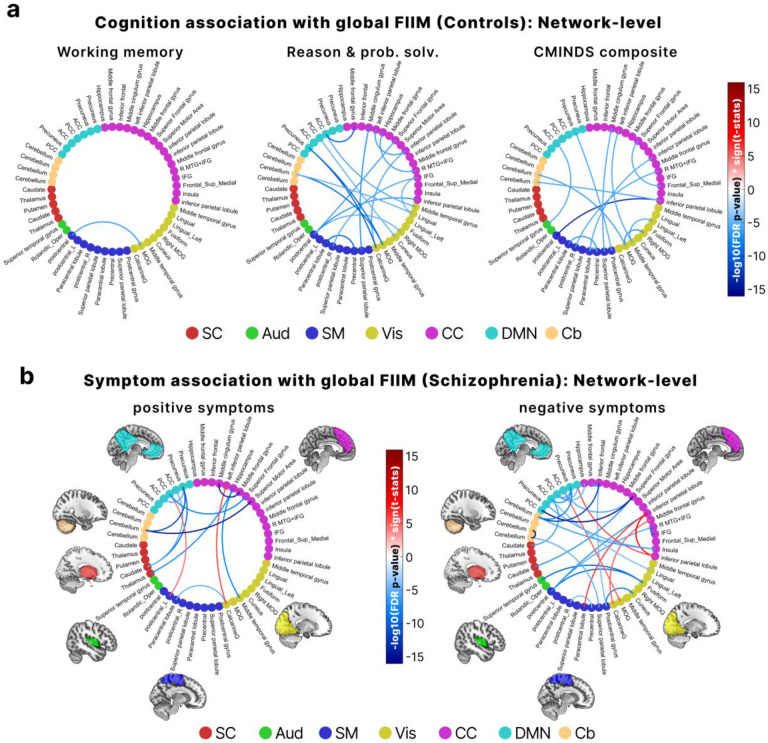
Circuit-level distribution of functional inertia links cognition and symptoms **(a)** Network-level associations between time-averaged whole-brain Functional Inertia Index Magnitude (FIIM) and cognitive performance in healthy controls. Significant associations are shown for working memory, reasoning, and problem solving, and the CMINDS composite score. Edges indicate interregional connections whose FIIM significantly relates to cognitive performance after FDR correction, colored by the direction and strength of association (signed −log_10_ FDR-corrected p-values). Node colors indicate large-scale functional systems, including subcortical (SC), auditory (Aud), sensorimotor (SM), visual (Vis), cognitive control (CC), default mode (DMN), and cerebellar (Cb) networks. Stronger functional inertia within distributed association, midline, and cerebellar circuits is associated with better cognitive performance. **(b)** Network-level associations between FIIM and clinical symptom severity in schizophrenia. Connections whose FIIM significantly relate to positive (left) and negative (right) symptom severity are shown. Most symptom-related associations involve midline default-mode hubs, limbic–thalamo–temporal pathways, fronto-striatal, cerebellar, and temporal–sensorimotor circuits, with stronger functional inertia predicting greater symptom severity in most connections. A smaller subset of sensory–association couplings exhibits the opposite relationship.

To formalize the idea that human cognition is shaped by context-dependent accumulation of experience, we adopt an inertial state-space formulation. At an abstract level, this describes how a person’s momentary internal state reflects a weighted history of prior states, with past configurations persisting and being differentially expressed depending on the current context. Here, we operationalize this constraint using brain data, treating large-scale neural activity as an observable proxy for the underlying cognitive state illustrated schematically in Fig. 1c. The formulation is observational, providing a compact mathematical description of how observations across time contribute to the present latent configuration.

Let *x*(*t*) denote the latent state of the system at time *t*, corresponding to the internal cognitive configuration depicted in the schematic model, and let *y*(*t*) denote the observed multivariate brain signal used to infer this state. In its most general continuous-time form, the inertial state-space model is defined as

(1) $$x\left(t\right)=\int_{0}^{t}B\left(t,\tau |u\left(t\right)\right)y\left(\tau \right)d\tau +D\left(t\right)u\left(t\right),$$

where *B*(*t, τ* | *u*(*t*)) is a context-modulated accumulation kernel specifying how prior observations *y*(*τ*) at time indices *τ* ≤ *t* contribute to the current state, and *u*(*t*) represents external inputs or contextual influences when present. The term *D*(*t*)*u*(*t*) captures direct effects of such inputs on the latent state.

Within this formulation, the accumulation kernel defines how present brain states are constructed from observations across time in a context-dependent manner. This captures how state evolution unfolds within a latent space shaped jointly by accumulated history and current context, with functional inertia emerging as the degree to which prior configurations constrain ongoing reorganization.

For discretely sampled neuroimaging data, such as fMRI, the formulation becomes

(2) $$x_{t}=\sum_{\tau =1}^{t}B\left(t,\tau |u_{t}\right)y_{\tau }+Du_{t},$$

where *x*_*t*_ denotes the latent state at time index *t*, and *y*_*τ*_ the observed signal at time indices *τ* ≤ *t*. An intuitive version of the ISSM formulation is provided in Fig. 1b.

This formulation is deliberately broad. While we focus here on brain-derived observations, the same inertial description applies to human cognition more generally, with different observational modalities, including neural or behavioral readouts, corresponding to different instantiations of *y*(*t*). Different accumulation kernels define alternative operationalizations of inertia, while preserving the constraint that present states reflect the cumulative trajectory leading to the present moment.

### 4.2. Zero-order instantiation of the ISSM

The inertial state-space formulation admits a zeroth-order instantiation in which the latent state is defined solely by accumulated observations, without requiring an explicit, parameterized input or task model. This regime arises naturally when state evolution is driven by internally generated dynamics or by contextual influences that are implicit or unmeasured, as in spontaneous thought, mind-wandering, memory recall, affective drift, or naturalistic conditions such as task-free viewing or listening. In these settings, the absence of explicit inputs does not imply a lack of structure; rather, it isolates a principled limit in which the influence of accumulated history on state evolution can be examined directly.

Resting-state fMRI provides a particularly clear and widely studied example of this regime. In the absence of externally imposed tasks, large-scale brain activity reflects internally generated fluctuations shaped by prior configurations of the system. For discretely sampled zero-order ISSM, we set *u*_*t*_ = 0 and equation (2) reduces to

(3) $$x_{t}=\sum_{\tau =1}^{t}B\left(t,\tau \right)y_{\tau }.$$

A natural and assumption-light choice in this zeroth-order setting is the uniform cumulative kernel,

(4) $$B\left(t,\tau \right)=\frac{1}{t},\;1\leq \tau \leq t,$$

which yields the ISSM latent representation

(5) $$x_{t}=\frac{1}{t}\sum_{\tau =1}^{t}y_{\tau }.$$

This choice does not assert that biological memory is uniform or non-decaying. Rather, it provides a minimal operationalization of inertia by defining the latent state as the running configuration implied by the trajectory to date, such that functional inertia is revealed by the deviation required for new observations to reorganize this configuration. By isolating state evolution from explicit inputs, the zeroth-order instantiation exposes inertia as an intrinsic property of brain dynamics while making the weakest possible assumptions about temporal weighting, task structure, or contextual drives, a choice that aligns naturally with resting-state conditions in which relevant influences are endogenous and unmeasured. More generally, alternative input-free accumulation kernels can be adopted within the same zeroth-order class to reflect different assumptions about temporal integration.

### 4.3. Functional Inertia and the Functional Inertia Index (FII)

Within the inertial state-space formulation, functional inertia corresponds to resistance to change in the latent state over time. We operationalize this constraint by quantifying the temporal rate of change of the latent trajectory, which we term the Functional Inertia Index. Because inertia reflects resistance to change, values of FII close to zero indicate strong inertia, whereas larger-magnitude values indicate active reconfiguration. Formally, the FII at time *t* is defined as the temporal derivative of the latent state,

(6) $$\text{FII}\left(t\right)=\frac{d}{\text{dt}}x\left(t\right),$$

or, in the discrete-time setting,

(7) $$\text{FI}I_{t}=x_{t}-x_{t-1}.$$

This definition holds regardless of how the latent state is constructed and therefore applies across different instantiations of the inertial state-space model.

The sign of the FII provides directional information about state evolution. Depending on the representational axis, positive FII may indicate low-inertia evolution toward a more coherent configuration, whereas negative values indicate low-inertia evolution toward a more divergent configuration. The magnitude of the FII, termed the Functional Inertia Index Magnitude, captures the strength of this deviation irrespective of direction. Importantly, the FII is not a standalone metric but an index derived directly from the inertial state-space formulation. It does not assume specific temporal scales, task structure, or mechanistic forces. Rather, it provides a principled, time-aware quantification of how strongly past configurations constrain present evolution. In this way, the FII serves as an operational lens through which functional inertia, as an organizing constraint of brain dynamics, can be measured and related to cognition and clinical phenotype.

### 4.4. Dynamic time warping instantiation of the ISSM

The inertial state-space formulation is agnostic to the representational axis used to define the observed trajectory *y*(*t*), as it can be applied to signals ranging from time-varying brain maps and regional activity to connectivity profiles or behavioral measures. Different choices of *y*(*t*) thus provide different operational views of the same inertial constraint. Here, we instantiate the ISSM using time-resolved interregional profiles that reflect the relational organization of large-scale brain function, in which dynamics emerge from evolving patterns of interaction. In this instantiation, we define the observed trajectory *y*(*t*) using dynamic time warping across brain region pairs^39,40^.

This choice is motivated by two considerations. First, brain dynamics unfold across multiple and nonuniform timescales, with intrinsic temporal variability that is not well captured by pointwise or fixed-lag comparisons; DTW explicitly accommodates such variability through time-adaptive alignment^21^. Second, DTW yields a distance measure that quantifies the magnitude of deviation between timescale-aligned interregional profiles^21,41^. These distances are non-negative, additive, and are defined as cumulative deformation costs across aligned time points, mirroring the algebraic structure of the accumulative ISSM. In the ISSM, observations are accumulated over time to form a latent trajectory and then differentiated to quantify resistance to reconfiguration; this accumulate–differentiate pipeline is most naturally interpreted when the observations themselves encode displacement. DTW, therefore, provides a natural operational basis for functional inertia, allowing resistance to change to be expressed as a force-like quantity derived from the cumulative deviation required to rewrite the system’s prior configuration.

In the DTW instantiation, the cumulative trajectory reflects interregional amplitude disparity^24^. When this trajectory decreases, disparity is being reduced; when it increases, disparity is growing. The signed FII therefore indicates the direction of reconfiguration (toward reduced versus increased interregional disparity), while its magnitude indicates how strongly the system is reconfiguring. In the DTW instantiation, the derivative of the cumulative disparity trajectory is sign-inverted so that positive FII corresponds to reconfiguration toward reduced interregional disparity and negative FII corresponds to reconfiguration toward increased disparity. The DTW latent trajectory is temporally smoothed before differentiation to reduce high-frequency noise^42^. The full algorithmic details of DTW distance computation, cumulative trajectory construction, parameter selection, and derivative estimation are provided in the Supplementary Methods.

#### Anchor baseline window:

Because the ISSM accumulates observations over time, estimates derived from the earliest frames are inherently unstable and sensitive to transient scanner effects^43^. To ensure a well-defined reference configuration, we define an anchor baseline window at the start of each scan, serving as a stable starting point for cumulative evaluation. This anchor does not imply a shared task or baseline state across individuals; rather, it provides a common reference against which subsequent observations are assessed, allowing us to determine whether new fluctuations reinforce or overcome the running configuration implied by prior activity. All implementation details related to anchor selection are provided in the Supplementary Methods.

### 4.5. fMRI data & processing

We analyze resting-state fMRI from the Function Biomedical Informatics Research Network (fBIRN) consortium. Data was acquired with a repetition time of 2 s and comprised 160 healthy controls (37.0 ± 10.9 yr, 45 F/115 M) and 151 individuals with schizophrenia (38.8 ± 11.6 yr, 36 F/115 M). Volumes underwent standard preprocessing such as slice-timing correction, rigid-body realignment, MNI spatial normalization, and 6 mm FWHM Gaussian smoothing^44^. Spatially independent components were then extracted using the NeuroMark ICA pipeline^23^, yielding 53 intrinsic connectivity networks (ICNs) common to all subjects. The resulting ICN time courses are detrended, despiked, and filtered using a Butterworth bandpass filter with a frequency cutoff of 0.01 − 0.15*Hz*^45^. The filter, with an optimal order of 7 as determined by MATLAB’s *buttord* function, maintained a passband ripple under 3 *dB* and a minimum stopband attenuation of 30 *dB*. Finally, we standardize the filtered ICNs through z-score transformation.

### 4.6. Recurrent functional inertia states estimation

fMRI whole-brain network inertia vectors across brain region pairs are clustered with standard *k*-means because of its widespread use in time-resolved fMRI analysis^28^. Time points across subjects are concatenated into a single time-by-feature matrix (time points as samples, edges as features) prior to k-means clustering. We implement the *k*-means algorithm with cluster numbers ranging from 1 to 10, setting the maximum number of iterations to 10,000 and using 20 random initializations to ensure robust convergence. The city-block (Manhattan) distance metric is selected for its demonstrated robustness in handling high-dimensional data^46^. The optimal cluster number is determined as 3 using the elbow criterion (See Supplementary material).

### 4.7. State metrics

Using the *k*-means clusters, we characterized each participant’s temporal behavior with three metrics. First, the mean dwell time (MDT) measures state persistence by averaging how long a participant remains in a state once it is entered^47^. Second, the fraction rate (FR) reflects overall occupancy by expressing the proportion of the total recording spent in each state^47^. Finally, the state-to-state transition probabilities form the transition matrix (TM), which quantifies the likelihood of a participant moving from one state to any other^47^.

### 4.8. Group analysis & regression models

We assess group differences between healthy controls and individuals with schizophrenia, associations with the Computerized Multiphasic Interactive Neurocognitive System cognitive scores, and associations with the Positive and Negative Syndrome Scale symptom score using generalized linear models (GLM) that included age, sex, scanning site, and mean frame displacement (FD) as covariates. For group difference analyses and CMINDS scores, a normal distribution is assumed, whereas a Poisson distribution is applied for PANSS score associations due to the positive integer and skewed and non-negative integer nature of the fBIRN dataset’s PANSS scores^24,48^. Multiple comparisons are controlled with the Benjamini-Hochberg false discovery rate (FDR) procedure.

### 4.9. Global FIIM & mediation analysis

To characterize whole-brain functional inertia, we summarized the functional inertia index magnitude by averaging it across time for each participant. This global FIIM reflects the overall strength with which past configurations constrain ongoing state evolution throughout the scan, with lower values indicating stronger functional inertia. We test whether the relationship between state-level dynamic metrics and symptom severity is mediated by the global FIIM using a single-mediator framework with covariate adjustment. Statistical significance is evaluated using nonparametric bootstrap resampling. Full mediation model specifications are provided in the Supplementary Methods.

## Supplementary Material

Supplementary Files

This is a list of supplementary files associated with this preprint. Click to download.

- 2026WiafeInertiasupplementary.docx

## Data Availability

The codes for all our analyses in MATLAB can be accessed through GitHub. The data was not collected by us and was provided in a deidentified manner. The IRB will not allow the sharing of data or individual derivatives, as a data reuse agreement was not signed by the subjects during the original acquisition.
